# Comparison between Fluorimetry (Qubit) and Spectrophotometry (NanoDrop) in the Quantification of DNA and RNA Extracted from Frozen and FFPE Tissues from Lung Cancer Patients: A Real-World Use of Genomic Tests

**DOI:** 10.3390/medicina57121375

**Published:** 2021-12-17

**Authors:** Katsuhiro Masago, Shiro Fujita, Yuko Oya, Yusuke Takahashi, Hirokazu Matsushita, Eiichi Sasaki, Hiroaki Kuroda

**Affiliations:** 1 Department of Pathology and Molecular Diagnostics, Aichi Cancer Center, Nagoya 4648681, Japan; esasaki@aichi-cc.jp; 2 Department of Respiratory Medicine, Kobe Central Hospital, Kobe 651115, Japan; jp.shirofujita@gmail.com; 3 Department of Thoracic Surgery, Aichi Cancer Center, Nagoya 4648681, Japan; yshima@aichi-cc.jp (Y.O.); y.takahashi@aichi-cc.jp (Y.T.); h-kuroda@aichi-cc.jp (H.K.); 4 Division of Translational Oncoimmunology, Aichi Cancer Research Institute, Nagoya 4648681, Japan; h.matsushita@aichi-cc.jp

**Keywords:** next-generation sequencing, PCR-based, UV absorbance measurement

## Abstract

*Background and Objectives:* Panel-based next-generation sequencing (NGS) has been carried out in daily clinical settings for the diagnosis and treatment guidance of patients with non-small cell lung cancer (NSCLC). The success of genomic tests including NGS depends in large part on preparing better-quality DNA or RNA; however, there are no established operating methods for preparing genomic DNA and RNA samples. *Materials and Methods:* We compared the following two quantitative methods, the QubitTM and NanoDropTM, using 585 surgical specimens, 278 biopsy specimens, and 82 cell block specimens of lung cancer that were used for genetic tests, including NGS. We analyzed the success rate of the genomic tests, including NGS, which were performed with DNA and RNA with concentrations that were outliers for the Qubit Fluorometer. *Results:* The absolute value for DNA concentrations had a tendency to be higher when measured with NanoDropTM regardless of the type of specimen; however, this was not the case for RNA. The success rate of DNA-based genomic tests using specimens with a concentration below the lower limit of QubitTM detection was as high as approximately 96%. At less than 60%, the success rate of RNA-based genomic tests, including RT-PCR, was not as satisfactory. The success rates of the AmpliSeqTM DNA panel sequencing and RNA panel sequencing were 77.8% and 91.5%, respectively. If at least one PCR amplification product could be obtained, then all RNA-based sequencing was performed successfully. *Conclusions:* The concentration measurements with NanoDropTM are reliable. The success rate of NGS with samples at concentrations below the limit of detection of QubitTM was relatively higher than expected, and it is worth performing PCR-based panel sequencing, especially in cases where re-biopsy cannot be performed.

## 1. Introduction

Recently, somatic mutations of *EGFR* and *BRAF* and gene rearrangements of *ALK* and *ROS1* have been recommended for testing before the initial treatment of patients with advanced non-small cell lung cancer (NSCLC) based on the guidelines from major professional organizations [1,2,3,4,5]. Furthermore, panel-based next-generation sequencing (NGS) has come into practice in daily clinical settings for diagnosis and treatment guidance. Preparing better quality DNA or RNA is more dependent on other pre-analytic procedures, such as what happens after the surgery, fixation period, fixation procedure in general (especially for the majority of the labs which work with formalin fixed paraffin embedded tissues), procedures in a pathology laboratory, and nucleic acid extraction protocols, which are included in the Standard PRE analytical Code (SPREC) [6]; thus, the accuracy of the measurement of DNA or RNA has also become more important. The preparation of a proper DNA or RNA extraction procedure and the establishment of an accurate quantification method of these samples play a key role in the success of these genomic tests; there are no established operating methods for preparing genomic DNA and RNA.

DNA and RNA have been quantified using spectrophotometry, historically [7,8,9]; however, three important clinical problems need to be clarified in this method. The first problem is that UV absorbance measurements are not selective for DNA, RNA, or protein. The second is that the absolute values vary widely with other contaminants and base composition. The third is that the accuracy of spectrophotometry tends to be inadequate at low concentrations of DNA and RNA. To overcome these drawbacks, fluorescence-based quantitation method, Qubit^TM^, has come to be widely used [10,11,12,13]. Quantitative polymerase chain reaction (qPCR) with a template-specific probe has also come into practice as an alternative quantification method. Although these two methods are certainly reliable, they are more laborious and expensive.

There are several reports that evaluated which methods are suitable for measuring DNA used for NGS. NanoDrop^TM^ tends to overestimate the concentration in several studies [14,15]. On the other hands, Hedyt C. et al. reported that there was no uniform tendency in the quantification methods of DNA concentration, and no difference in mutation analysis according to the results of the quantification method found, which included NanoDrop^TM^, Qubit^TM^, and qPCR [16].

Thus, no definitive quantification methods have been established and evaluated, especially in clinical practice. Here, we compared two commonly used quantitation methods, the Qubit^TM^ and NanoDrop^TM^, using clinical samples that were used for genetic tests, including NGS.

## 2. Materials and Methods

### 2.1. Samples

A total of 585 surgical specimens, 278 biopsy specimens, and 82 cell block specimens of lung cancer were selected from the registry of Aichi Cancer Center Hospital from January 2017 to December 2017. This study was approved by the Research Ethics Committee of Aichi Cancer Center (No.2020-2-34).

### 2.2. Extraction of DNA and RNA

Tumor samples were obtained at surgery and were rapidly frozen in liquid nitrogen. Frozen tumor tissues specimens were grossly dissected, and DNA and total RNA were isolated using a QIAamp DNA Mini Kit^TM^ and an RNeasy Mini Kit^TM^ (Qiagen, Valencia, CA, USA), respectively. Immediately after the biopsy, cytological specimens applied to an uncoated slide were immersed in 95% alcohol, and DNA and total RNA were isolated using a QIAamp DNA Mini Kit^TM^ and an RNeasy Mini Kit^TM^ (Qiagen, Valencia, CA, USA), respectively. 

DNA extraction from FFPE cell block specimens was performed using proteinase K. Briefly, the tissues were dewaxed with xylene and digested overnight at 56 °C and for 3 min at 95 °C with a 45:5:1 solution of RNase-free water, TaqGOLD^TM^ buffer, and proteinase K, respectively (50 µL > for surgery specimens and 30 µL > for biopsy specimens). After digestion, the DNA was eluted with RNase-free water (50 µL > for surgery specimens and 30 µL > for biopsy specimens). 

### 2.3. Quantification of DNA and RNA

The concentrations of DNA were determined using a Qubit 4^TM^ Fluorometer with dsDNA HS Assay Kit^TM^ for Qubit and a NanoDrop™ Lite Spectrophotometer (Thermo Fisher Scientific, Wilmington, DE, USA). 

### 2.4. Routine Genomic Tests

Genomic tests for *EGFR*, *KRAS*, *BRAF*, *HER2,* and *ALK* were carried out as previously reported [17]. Briefly, EGFR gene, *KRAS* gene, and *BRAF* gene were analyzed by Cycleave^TM^ PCR and direct sequencing method. *HER2* gene was analyzed by fragment PCR. An immunohistochemical analysis of anaplastic lymphoma kinase (ALK) with a mouse monoclonal antibody to ALK (ALK1, Dako) was performed. 

### 2.5. Panel Sequencing

DNA- and RNA-based panel sequencing was performed only if one PCR amplification product could be obtained in routine genomic tests. Sequencing was performed using Ion 540 chips on Ion Torrent S5 Sequencer™ using barcoded libraries prepared with AmpliSeq™ Library Preparation Kits (Thermo Fisher Scientific, Wilmington, DE, USA) according to the manufacturer’s protocols. We defined the success of NGS as more than 1000 mean depth.

### 2.6. Statistical Analysis

Statistical analysis was performed using the regression analysis between the concentration evaluated with Nonodrop™ and Qubit™. All statistical analyses were performed using the JMP 12 software (SAS Institute, Cary, NC, USA).

## 3. Results

### 3.1. Quality of Frozen-DNA and RNA

**Figure 1:**
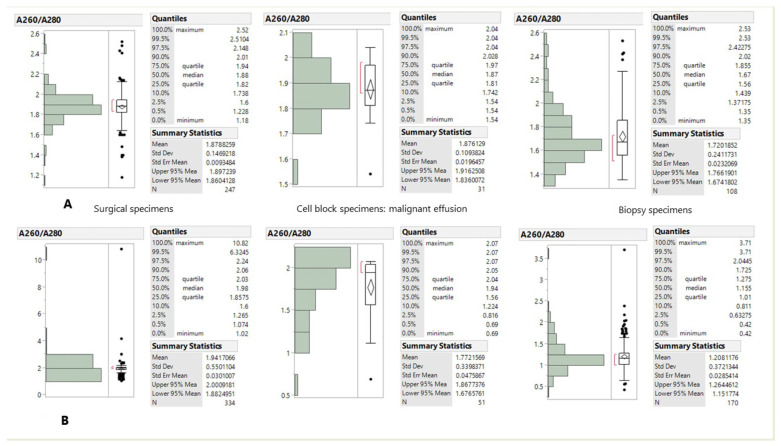
DNA (**A**) and RNA (**B**) quality measured by A260/A280 and grouped according to tissue type.

The purity of DNA and RNA determined by the absorbance ratio at wavelength 260/280 nm (A260/A280) is shown in Figure 1. The median value of A260/A280 for DNA was 1.88 for the surgical specimens, 1.67 for the cell block specimens and 1.67 for the biopsy specimens (Figure 1A). The median value of A260/A280 for RNA was 1.98 for the surgical specimens, 1.94 for the cell block specimens, and 1.155 for the biopsy specimens (Figure 1B). 

### 3.2. Quantification of Frozen DNA by NanoDrop and Qubit

**Figure 2:**
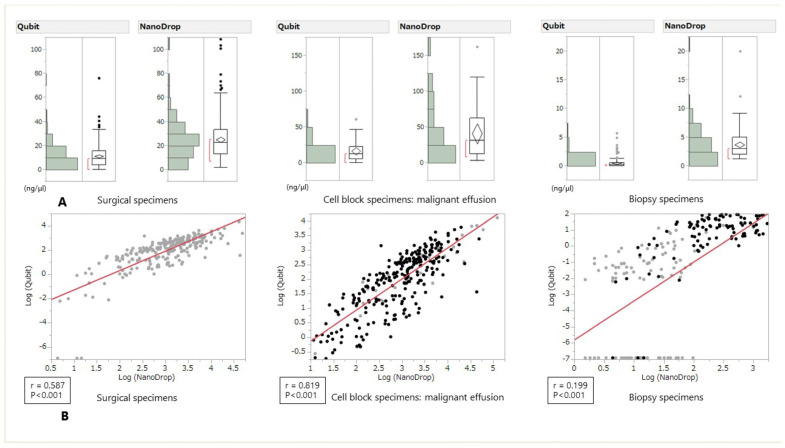
DNA concentration measured by Qubit and NanoDrop and grouped according to tissue type (**A**): Surgical specimens, Cell block specimens, Biopsy specimens and Scatter plot for the correlation analysis between Qubit and NanoDrop measurements (**B**). The R coefficients were 0.587 for the surgical specimens, 0.819 for the cell block specimens, and 0.199 for the biopsy specimens.

The comparison of DNA concentrations quantified by NanoDrop and Qubit is shown in Figure 2. The absolute value of DNA concentration had a tendency to be higher when measured with NanoDrop regardless of the type of specimen. In the regression analysis, a positive correlation was found in this order: cell block specimens, surgical specimens, and biopsy specimens (Figure 2).

### 3.3. Quantification of Frozen RNA by NanoDrop and Qubit

**Figure 3:**
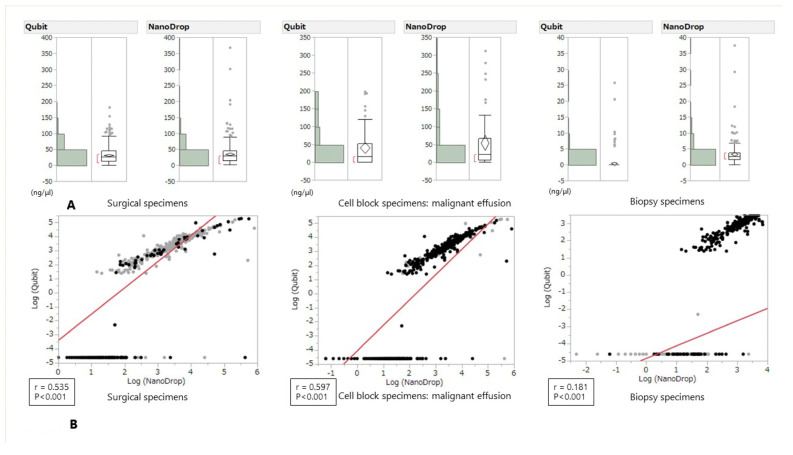
RNA concentration measured by Qubit and NanoDrop and grouped according to tissue type (**A**): Surgical specimens, cell block specimens, Biopsy specimens) and Scatter plot for the correlation analysis between Qubit and NanoDrop measurements (**B**). The R coefficients were 0.535 for the surgical specimens, 0.597 for the cell block specimens, and 0.1 for the biopsy specimens.

The comparison of RNA concentrations quantified by NanoDrop and Qubit is shown in Figure 3A. Although the absolute value of RNA concentration had a tendency to be slightly higher when measured with NanoDrop regardless of the type of specimen, several opposite cases were also observed. In the regression analysis, a positive correlation was also found in the following order: cell block specimens, surgical specimens, and biopsy specimens; however, the correlation coefficient tended to be slightly weaker than that for DNA (Figure 3).

### 3.4. Results of Routine Genomic Tests with Suboptimal DNA and RNA Specimens

The characteristics of the tissue used for genomic tests are shown in Table 1. The success rates of routine genomic tests using DNA or RNA with concentrations that are outliers when measured with Qubit are shown in Table 2. The success rates of the DNA-based genomic tests, including Cycleave^TM^ PCR and fragment PCR, were as high as approximately 96%. At less than 60%, the success rate of the RNA-based genomic test, including RT-PCR, was not as satisfactory.

### 3.5. Results of Routine NGS Panel Sequencing with Suboptimal DNA and RNA Specimens

Panel sequencing was performed in cases where at least one routine genomic test was successful. The success rate of NGS panel sequencing using DNA or RNA with outlier concentrations calculated with Qubit is as follows: the success rates of the AmpliSeq^TM^ DNA panel sequencing and RNA panel sequencing were 77.8% (14/18) and 91.5% (43/47), respectively. The minimal requirement for the amount of DNA and RNA for panel sequencing is 10 ng; however, the success rate of both NGS sequencing runs was higher than expected.

## 4. Discussion

In this study, we showed that DNA concentration had a tendency to be higher when measured with NanoDrop^TM^ regardless of the type of specimen; however, even though slightly higher RNA concentrations were measured with NanoDrop, regardless of the type of specimen, several opposite cases were also observed. Twenty-eight biopsy specimen and one pleural effusion sample showed opposite results. It may be due to the equipment characteristics that the lower the concentration measured by Nanodrop ^TM^, the more the measured value varies, and that the Qubit ^TM^ has a measurement limit value. The success rate of routine DNA-based genomic tests, including NGS, with samples with suboptimal conditions that had outlier concentrations as calculated with Qubit was approximately 80%. The success rate of the RNA-based method was approximately 50%. The minimal requirement of the amount of DNA and RNA for panel sequencing is 10 ng; however, the success rate of both NGS sequencing runs was higher than expected. The correlations of the two methods in different samples were hugely different (Figure 2 and Figure 3). These differences may be due to the small amount of RNA or the amounts of contaminants in the DNA extraction method. However, tumors harboring driver mutations may also increase RNA expression of the driver gene, which may lead to more successful rate of genomic tests than expected.

There are few studies comparing DNA and RNA quantification methods from FFPE samples, and the results do vary [15]. Hadd et al. [18] used NanoDrop^TM^ for DNA quantification for targeted NGS. On the other hand, Qubit^TM^ is reported to be commonly used as the easiest, most reliable, and cost-effective quantification method for NGS [19]. Simbolo M et al. have proposed that the recommended workflow for quantifying DNA extracted from FFPE tumor tissues suitable for NGS is, first, to evaluate the quality of the sample with a NanoDrop^TM^, and subsequently, to quantify the concentrations of the sample with Qubit^TM^ [15]. Heydt C. et al. showed that Qubit^TM^ and qPCR can be used for downstream applications and even the NanoDrop^TM^ can be used for subsequent sample analysis with massively parallel sequencing [16]. Some studies have reported that qPCR is the most reliable method and spectrophotometric analysis is the least reliable; however, it is time-consuming, expensive, and not practical for routine laboratory tests with a high sample throughput, which has also been stated by other studies [14,20].

The most striking difference between using Qubit^TM^ and NanoDrop^TM^ is the accuracy of the Qubit^TM^ assays which provide much more reliable information than NanoDrop^TM^. The measurement error when quantifying samples of 10 ng/µL of DNA with Qubit^TM^ is within 1% and 5% when using NanoDrop^TM^ [20,21,22,23]. In terms of accuracy, qPCR is the ideal quantifying method of DNA used for NGS [24]. However, qPCR takes much more time and is more expensive than spectrophotometric analysis. In terms of sample loss, for NanoDrop^TM^ and Qubit^TM^ analysis, only small sample volumes of 1–2 µL are required, which means fewer samples are used for quantitation and more samples are available for genomic analysis including NGS. 

Considering the success rate, the DNA-based AmpliSeq^TM^ NGS is worth performing, because its success rate is high even when the DNA concentration is outside the measurement range. On the other hand, in the case of RNA, if even one PCR amplification product can be obtained, it is worth performing panel sequencing, regardless of the RNA concentrations. In the case of commercial-based NGS panel sequencing, the minimum requirements for tissue volume and tumor contents were 25 mm^3^ and 20% in FoundationOne CDx^TM^ [25], respectively, and the required minimum values for the Oncomine™ Dx Target Test are shown in Table 3 [25]. In these situations, it seems very important to show the success rate of a panel-based NGS test (Table 4).

Limitations of this study include that the DNA and RNA quality such as the DIN and RIN value and A260/230 ratio were not measured. For example, paraffin-embedded tissues are likely to cause sequence breaks, and long fragment samples are not easy to detect. These points may be solved by measuring the DIN and RIN value.

## 5. Conclusions

In conclusion, DNA concentration had a tendency to be higher when measured with NanoDrop^TM^; however, this was not the case for RNA. Although DNA-based AmpliSeq^TM^ NGS when the DNA is outside the measurement rate does not come from the FFPE tissue which is the main problem in most diagnostic labs, the success rate of NGS with low sample concentration measured with both methods was relatively higher than expected, and it is worth performing PCR-based panel sequencing, especially when rebiopsy is difficult to perform. This result may be due to the high expression of the mutation-derived gene used in tumor cells, suggesting the usefulness of using RNA for NGS.

## Figures and Tables

**Table 1:** Characteristics of the tissues used for genomic tests.

Tissue Type	DNA (%)	RNA (%)
Surgery specimen	249 (64.3)	336 (60.2)
Cell block specimen	31 (8.1)	51 (9.2)
Biopsy specimen	107 (27.6)	171 (30.6)
Total	387 (100)	558 (100)

**Table 2:** Success rate of routine genomic tests using DNA or RNA with concentrations that are outliers when calculated with Qubit.

DNA Based Genomic Tests	Success Rate (%)
Cycleave^TM^ *EGFR* L858R	26/27 (96.3)
Fragment *EGFR* exon 19 deletions	26/27 (96.3)
Cycleave^TM^ *EGFR* exon 20 insertions	25/27 (92.6)
Cycleave^TM^ *KRAS* G12X	26/27 (96.3)
Fragment *HER2* exon 20 insertions	26/27 (96.3)
RNA based genomic tests	
*MET* exon 14 RT-PCR	86/153 (56.2)
*EGFR* RT-PCR	54/150 (36.0)
*KRAS* RT-PCR	91/150 (60.7)
*TP53* RT-PCR	60/144 (41.7)
*ALK* fusion RT-PCR	59/150 (39.3)
*BRAF* RT-PCR	78/148 (52.7)

**Table 3:** Required sample concentrations and *R^2^ values.

Sample Type	Required Concentration	Required *R^2^ Values
DNA	≥0.83 ng/µL	≥0.99
RNA	≥1.43 ng/µL	≥0.98

*R^2^ values should be evaluated only if the standard curve includes 3 or more points.

**Table 4:** Success rate of NGS panel sequence using DNA or RNA with concentrations that are outliers when calculated with Qubit.

Methods	Success Rate (%)
AmpliSeq^TM^ DNA panel sequence	14/18 (77.8)
AmpliSeq^TM^ RNA panel sequence	43/47 (91.5)

## Data Availability

Data are available upon reasonable request to the Corresponding author.
